# Analysis of the Impact of Atmospheric Models on the Orbit Prediction of Space Debris

**DOI:** 10.3390/s23218993

**Published:** 2023-11-06

**Authors:** Yigao Ding, Zhenwei Li, Chengzhi Liu, Zhe Kang, Mingguo Sun, Jiannan Sun, Long Chen

**Affiliations:** 1 Changchun Observatory, National Astronomical Observatories Chinese Academy of Sciences, Changchun 130117, China; 2 University of Chinese Academy of Sciences, Beijing 100049, China; 3 Key Laboratory of Space Object and Debris Observation, Purple Mountain Observatory, Chinese Academy of Sciences, Nanjing 210008, China

**Keywords:** atmospheric model, orbit determination, orbit prediction

## Abstract

Atmospheric drag is an important influencing factor in precise orbit determination and the prediction of low-orbit space debris. It has received widespread attention. Currently, calculating atmospheric drag mainly relies on different atmospheric density models. This experiment was designed to explore the impact of different atmospheric density models on the orbit prediction of space debris. In the experiment, satellite laser ranging data published by the ILRS (International Laser Ranging Service) were used as the basis for the precise orbit determination for space debris. The prediction error of space debris orbits at different orbital heights using different atmospheric density models was used as a criterion to evaluate the impact of atmospheric density models on the determination of space-target orbits. Eight atmospheric density models, DTM78, DTM94, DTM2000, J71, RJ71, JB2006, MSIS86, and NRLMSISE00, were compared in the experiment. The experimental results indicated that the DTM2000 atmospheric density model is best for determining and predicting the orbits of LEO (low-Earth-orbit) targets.

## 1. Introduction

Atmospheric drag is an important influencing factor in determining and predicting the orbits of low-orbit space debris. Density distribution is a key physical quantity in studies of the variation laws of satellite motion under the influence of atmospheric drag. The so-called atmospheric density model is a mathematical model that calculates atmospheric density and its changes at corresponding time positions based on relevant parameters [1,2,3,4].

At present, some of the most commonly used atmospheric density models for space debris orbit determination and prediction include the Jacchia series, the Mass Spectrometer Incoherent Scatter Radar (MSIS) series, the Drag Temperature Model (DTM) series, and so on [5]. MSIS is an empirical atmospheric density model series that combines approximate neutral gas particle density, temperature, and solar radiation flux (F10.7) values, and values describing the geomagnetic activity (Ap) levels. The mathematical model of this mode is a spherical harmonic suitable for remote observation with multiple satellites [6,7,8,9,10]. The Jacchia model series is an atmospheric model established using satellite orbital decay data, and a series of other models have been developed based on this foundation, such as Jacchia–Bowman 2006. The basis for the Jacchia–Bowman 2006 (JB2006) atmospheric density model is the COSPAR International Reference Atmosphere 72 (CIRA72) atmospheric density model. The JB2006 model uses a new half-year density equation to replace the old equation in the Jacchia model series with a new solar index. In addition, several other equation-modeling methods have been incorporated in the JB2006 model to correct daily variations in errors [11,12,13,14]. DTM is a semi-empirical model that describes the temperature, density, and composition of the Earth’s thermosphere. The earliest DTM model is based on the Jacchia model, which more accurately represents the atmospheric density under extreme solar and geomagnetic conditions compared to the Jacchia model. The DTM model series is the result of simulation under moderate solar conditions, making it suitable for various applications [15,16,17].

There are two main ways to evaluate the performance of the above density modes: first, comparative analysis at the density level; second, analysis of the application level of orbit prediction [18]. Qiu Hongxing analyzed the impact of eight commonly used atmospheric density models on orbit prediction accuracy using GPS data [19]. Liu Wei and others used GPS data from the Tiangong Space Station to analyze the impact of atmospheric density models under different geomagnetic and solar radiance intensities on orbit prediction accuracy [20].

In the application of space target orbit determination, we found that some atmospheric density models do not seem to be ideal. When using satellite laser ranging data released by the ILRS [21] for orbit determination, some atmospheric density models are unable to perform orbit determination. In the case of using certain atmospheric models, the process of iteratively fitting observation data for orbit prediction in orbit determination calculations does not converge, making it impossible to complete orbit determination calculations. Therefore, an idea emerged to quantitatively compare the advantages and disadvantages of different atmospheric density models. Based on previous research [22,23,24,25], this method uses satellite laser ranging data published by the ILRS for orbit determination. This method evaluates the impact of different atmospheric density models on orbit prediction by using several specific space targets to represent retired satellites in space debris types. In contrast to the GPS data mentioned above, the laser ranging data of the experimental target can be easily obtained from the official website of the ILRS. By comparing orbital prediction errors, we can study the impact of different atmospheric density models on orbital prediction accuracy.

## 2. Basic Methods

We performed the orbit determination of space targets in this experiment using the following methodology [26,27]:

(1) Obtain measurement data;
(2) Preprocess measurement data to eliminate outliers;
(3) Set parameters for each dynamic model;
(4) Obtain the initial position velocity, and bring it into the mechanical model to obtain the acceleration for orbit integration calculations;
(5) Obtain orbit prediction at the target time, incorporate observation values, and use appropriate algorithms for iterative calculations. In this experiment, the least squares method was applied to calculate the correction using observed data;
(6) Calculate the difference between the position vector obtained from the iteration of the epoch time and the observation data. Determine whether the difference is less than the preset convergence limit, and, if it is less, the orbit determination calculation is completed. It is also possible to determine whether the orbit determination calculation is completed by iteratively calculating the correction value. If the correction value is less than the preset convergence limit, the orbit determination calculation is completed. This experiment uses correction values to determine convergence.

The basic steps for orbit prediction in this experiment are as follows:

(1) Obtain the initial range velocity (IRV). In this experiment, IRV refers to the position and velocity parameters of the experimental target at the beginning of the prediction;
(2) Bring this IRV into the mechanical model to calculate the acceleration value of targets at this epoch;
(3) Incorporate the position parameters, velocity parameters, and acceleration values into the orbit integrator to calculate the position and velocity parameters 30 s afterward. This experiment uses an Adam Cowell integrator with an integration period of 30 s;
(4) From step 3, we can obtain new position and velocity parameters. Introduce new positional velocity parameters into the dynamic model to obtain the acceleration values at the new epoch;
(5) Incorporate the position parameters, velocity parameters, and acceleration values obtained from step 4 into the orbit integrator to calculate position and velocity parameters 30 s afterwards;
(6) Repeat steps 4 and 5 until the target time position is reached.

In the above orbit determination and prediction calculations, atmospheric drag is an important component of the dynamic model. The atmospheric density is an important parameter for calculating atmospheric drag; therefore, the atmospheric density model affects the orbit determination and prediction of space debris in low Earth orbit.

In this way, the errors caused by different atmospheric density models will continue to accumulate and amplify.

There are currently not many low Earth orbit space debris observations with high-precision observation data. As one of the sources of space debris, scrapped satellites are used to represent space debris in this study. Laser ranging data are an important component of high-precision observation data for space targets. Therefore, this study selected satellite data publicly released by ILRS as the experimental object. Although the experimental target has not yet been scrapped, it has the same operational logic as space debris when these targets do not transfer their orbits. In this experiment, three days of laser ranging data were used to determine and predict orbits. This ensured that the target to be calculated had sufficient data points. Some low-orbit targets have limited observation data points, making it difficult to determine their orbits with daily data. Observation outliers need to be removed before orbit determination. In this experiment, we used a simplified dynamic model (SGP4) for orbit prediction to screen out observation data with errors greater than the preset limits. Orbit determination calculation is a continuous iterative fitting calculation that utilizes observation data and orbit prediction results. For low-Earth-orbit satellites, if the time span of the observation data is too large, the accumulated error in orbit prediction will be very large. When performing orbit determination iteration calculations, the fitting calculation results will not converge. If the reference observation data time span is too long, it will result in a large quantity of valid data being removed as outliers. Therefore, we ultimately decided to choose three days of observation data. For example, using data from 1 January to 3 January 2015, orbit determination and prediction were carried out to obtain the space debris prediction velocity and position parameters (P) from 0:00 on 5 January 2015 to 0:00 on 6 January 2015, UTC time. We then compared the orbit prediction results, P, with the orbit determination results from January 5th, D, and calculated the ERROR value (unit: m) as the evaluation value for the different atmospheric density models. The ERROR equation is as follows:

(1) $$ERROR=\sum_{T_{24}}^{T_{48}}\sqrt{{\left(D_{X}-P_{X}\right)}^{2}+{\left(D_{Y}-P_{Y}\right)}^{2}+{\left(D_{Z}-P_{Z}\right)}^{2}}/1441,$$

In this equation, D is the result of orbit determination, and P is the position parameter of the orbit prediction. In this experiment, one data point was obtained every minute. Therefore, there were a total of 1441 data points for orbit prediction from 24 h to 48 h. The larger the value obtained, the lower the accuracy.

To quantitatively evaluate the impact of different atmospheric density models on the space debris orbit determination calculation, we propose the following method: extrapolate the average accuracy of the prediction error from 24 to 48 h, calculated using different atmospheric density models for target space debris at different altitudes, from high to low. The scoring method is to rank the prediction errors of different density models in the same time period from small to large. Because this article considers a total of eight models, the score is inversely proportional to the error ranking with a score from 8 to 1. The highest score is 8 points, and the lowest score is 1 point. Thus, by adding up the accuracy scores of the same target debris and atmospheric densities throughout the year, we can obtain emission scores ranging from high to low for different orbital altitude and atmospheric density models. Based on this series of scoring results, a quantitative evaluation can be performed based on the impact of different atmospheric density models on the orbit determination calculations.

## 3. Selection of Experimental Data

Taking the DTM atmospheric model as an example, the density of the thermosphere atmosphere at an altitude of 120–1500 km can be calculated using the following equations [16,28]:

(2) $$f_{i}(z)={\left[\frac{T_{120}}{T(z)}\right]}^{1-\alpha +\gamma_{i}}exp(-\sigma \gamma_{i}\zeta ),$$

(3) $$\rho (z)=\sum_{i}\rho_{i}(120\;km)f_{i}(z)exp(G_{i}(L))$$

where $T(z)=T_{\infty }-(T_{\infty }-T_{120})exp(-\sigma \zeta )$.$T_{\infty }$ is the outer atmospheric temperature, α is the diffusion coefficient of He and H, $\gamma_{i}=m_{i}g(120\;km)/(\sigma kT_{\infty }),m_{i}$ is the atomic or molecular mass of the component, g(120 km) is the gravitational acceleration at 120 km altitude, σ is the vertical temperature gradient, k is the Boltzmann constant, ζ is the altitude, $\rho_{i}(120\;km)$ is the density of component i at an altitude of 120 km, and $G_{i}(L)$ is used to describe periodic and nonperiodic changes. Periodic changes are defined as annual and semiannual terms, as well as diurnal, semidiurnal, and terdiurnal terms [16].

**Figure 1:**
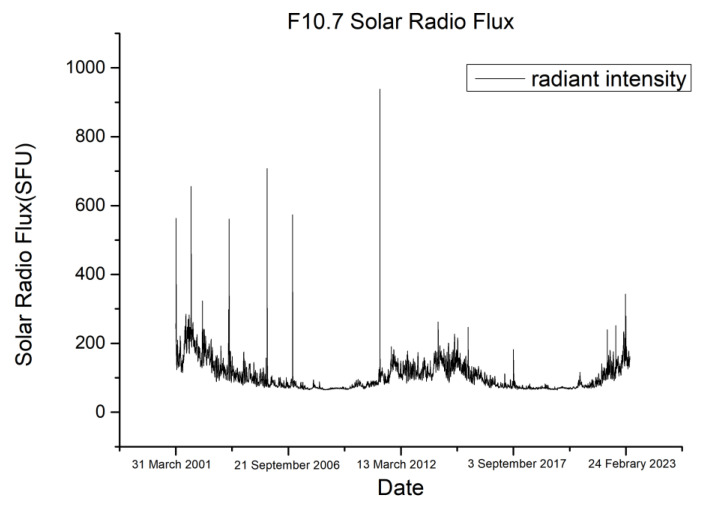
F10.7 solar radio flux versus time.

The above equations show that the solar radio flux and the geomagnetic index are important factors affecting the atmospheric density. F10.7 is the solar radiation flux at a wavelength of 10.7 cm (2800 MHz), which can well describe the radiation level of the Sun [28,29,30,31,32,33,34,35]. Figure 1 shows the time variation of the F10.7 radiation intensity; the data are from the public data of the National Oceanic and Atmospheric Administration [36].

Figure 1 shows that the solar flux intensity reached a peak in 2015. The solar intensity changed dramatically in that year, so we used the 2015 data for this experiment. This experiment used space debris laser ranging data publicly released by the ILRS as the basis for orbit determination and prediction. Target selection should strive to cover different orbital altitudes. Based on the above requirements, the target space debris selected for this experiment is described in Table 1 based on public data [37,38].

For low-orbit space debris, atmospheric drag is the most important non-conservative perturbation force affecting its orbit, and its calculation method is as follows:

(4) $$\vec{F}=-\frac{1}{2}\rho C_{D}Av_{r}^{2}\vec{e_{vr}}$$

In the equation, $C_{D}$ is the drag coefficient of the space debris, A is the windward area of the space debris perpendicular to the direction of the motion velocity, $v_{r}$ is the velocity of the space debris relative to the atmosphere, $\vec{e_{vr}}$ is the unit vector of $v_{r}$, ρ is the atmospheric density. Therefore, the acceleration generated by atmospheric resistance can be expressed as:

(5) $$\vec{a_{r}}=-\frac{1}{2}\rho C_{D}\frac{A}{m}v_{r}^{2}\vec{e_{vr}}$$

In the equation, $\frac{A}{m}$ is the area-mass ratio, which is a parameter describing the physical characteristics of the space debris and an important parameter for calculating the impact of atmospheric drag on the space debris.

The data volume of these five satellites is relatively sufficient, and their geometric shapes are relatively simple. They cover a track altitude ranging from 450 km to 1500 km, and their mission time range just covers the solar variation peak in 2015. The processing results of these five satellites can represent the space debris, which is the retired satellites with small area-mass ratios.

The perturbation force of space debris in low Earth orbit can be divided into two categories: the conservative forces and the non-conservative forces. The conservative forces include the gravity of the Earth; the gravity of the Sun, the Moon, and other celestial bodies; the solid tide and ocean tide perturbation; and the relativistic perturbation. The non-conservative forces include the atmospheric resistance, the solar light pressure, the Earth-shine radiation pressure, etc. [39,40,41]. The influence of the atmospheric model explored on orbit determination in this experiment is a non-conservative force perturbation. To control the variables, the same model is used for all other perturbations.

- The 70-order JGM3 Earth gravity field model is used for the Earth gravity calculation [42];
- The planetary ephemeris DE200 provided by the Jet Propulsion Laboratory (JPL) of the United States is used to calculate the gravity of the solar, lunar, and other celestial bodies [43];
- The TOPEX 3.0 model is used for ocean tide perturbation calculation, and the solid tide calculation is represented by the coefficient change in the spherical harmonics of the Earth’s gravity field [26,27];
- The relativistic perturbation can be calculated using the following equation [44]:
  (6) $$\ddot{\vec{r}}=-\frac{GM}{r^{2}}\left(\left(4\frac{GM}{c^{2}r}-\frac{v^{2}}{c^{2}}\right)\vec{e_{r}}+\frac{4v^{2}}{c^{2}}\left(\vec{e_{r}}\cdot \vec{e_{v}}\right)\vec{e_{v}}\right),$$
- The non-conservative force solar light pressure and the Earth radiation pressure are related to the Sun–Earth position and the solar flux intensity; This study computes the Earth radiation pressure according to a Ph.D. dissertation by Knocke P, 1989 [45]; The solar radiation pressure in this study is modeled by the following equations [46]:
  (7) $$\begin{array}{cc} \ddot{\vec{a_{srp}}}=k*({CR}_{0} & +\;{CR}_{1}*(t-t_{0})+{CR}_{2}*{\left(t-t_{0}\right)}^{2})*Ps*Area-to-mass\\ & \;-ration*\frac{{Au}^{2}}{r^{2}}*\vec{r}, \end{array}$$
  where,
  k is the Earth shadow factor;
  $\vec{r}$ is the unit vector from the satellite to the Sun;
  ${CR}_{0}$, ${CR}_{1}$, ${CR}_{2}$ and the area-to-mass-ratio can be treated as the parameters related to the space target;
  Au is the astronomical unit in meters;
  r is the distance between the satellite and the Sun in meters;
  Ps is the solar radiation pressure near the Earth;
- The Cowell numerical integration method is adopted for integration calculation, and the calculation is carried out in 30 s steps;
- The initial orbit state vector calculated for orbit determination includes the position vector and the velocity vector at the initial time, both of which are calculated according to the TLE (tow line element) published by NORAD.

## 4. Data-Processing Results

The data disclosed by the ILRS included data from multiple satellite laser ranging stations. Orbit determination and prediction were performed on a single satellite over three days of multi-station data. We evaluated and calculated the ERROR value according to the method described in Section 2. Taking the 2015 data processing results of the SpinSat satellite as an example, the orbit prediction errors of the different atmospheric density models are described in Table 2.

The orbit altitude of the SpinSat satellite is 425 km. The first column in the table is the time of the root mean square error (RMSE). Next, each column shows the prediction errors of the different atmospheric density models. The unit of error is in meters. In the table, “none” represents a failure to successfully determine the orbit using the model.

According to the method described in Section 2 and the prediction error results in Table 2, the error ranking table obtained is as in Table 3.

By calculating the scores in each column, we determined the advantages and disadvantages of the orbital prediction applications for the different atmospheric density models at a 425 km orbital altitude in 2015.

Table 4 shows the calculation results of the laser ranging data for all of 2015. The columns represent different atmospheric density models, and each row represents satellites with different orbital altitudes. Using the 2015 global station laser ranging data for orbit determination and prediction for different atmospheric density models, Table 4 shows the quantitative evaluation ranking scores of the atmospheric density models with different orbital altitudes based on the evaluation method proposed in Section 2. The scores for each grid in Table 4 are the sum of the corresponding model’s application scores for the orbit prediction throughout 2015. Taking the corresponding score of the DTM78 column in the Spinsat row as an example, this score is the sum of all the scores in the DTM78 column in Table 3.

From Table 4, it is not difficult to see that the different atmospheric density models have different impacts on the accuracy of orbit determination and prediction for satellites with different orbital altitudes. The representative accuracy calculation results from the 2015 data are taken and plotted below.

**Figure 2:**
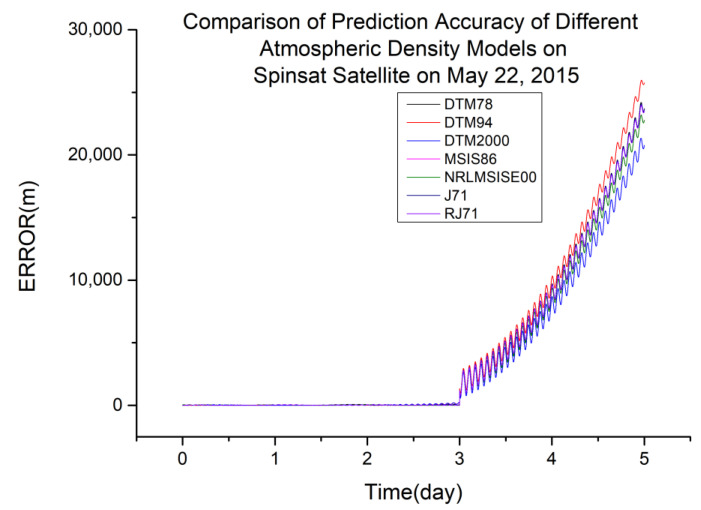
Comparison of the prediction accuracies of different atmospheric density models using the SpinSat satellite from 22 May 2015.

In Figure 2, the vertical axis represents the prediction error of different atmospheric density models in meters, and the horizontal axis is the time since 00:00 on the first day of observation data in days. Based on the data in Table 4, DTM2000 and RJ71 are more suitable for orbit determination and prediction calculated using global laser ranging data at an orbital altitude of 400 km.

**Figure 3:**
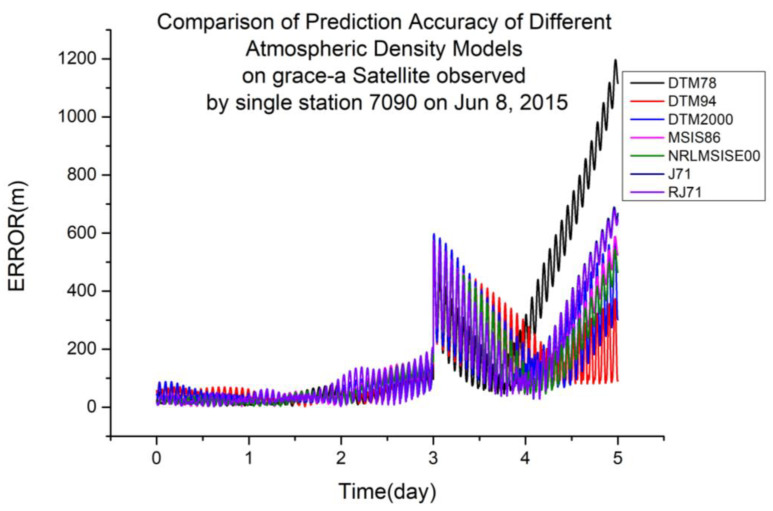
Comparison of the prediction accuracies of different atmospheric density models using the GRACE-A satellite on 5 July 2015.

As shown in Table 4, NRLMSISE00 is more suitable for orbit determination and prediction calculated using global laser ranging data at an orbital altitude of 485 km. Figure 3 shows a typical scenario.

**Figure 4:**
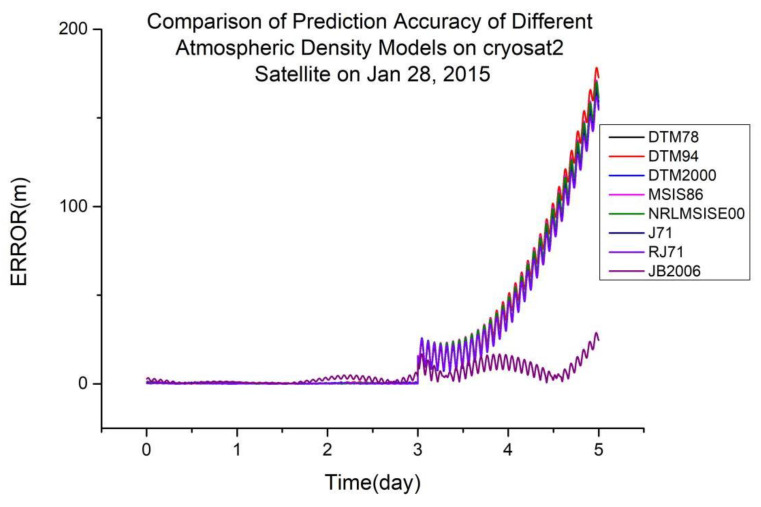
Comparison of the prediction accuracies of different atmospheric density models using the CryoSat2 satellite on 28 January 2015.

As shown in Table 4, JB2006 is more suitable for orbit determination and prediction calculated using global laser ranging data at an orbital altitude of 720 km. Figure 4 shows a typical scenario.

**Figure 5:**
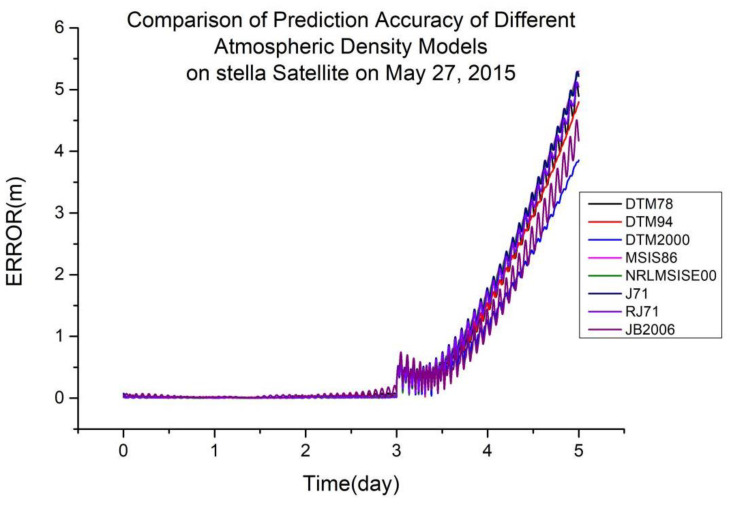
Comparison of the prediction accuracies of different atmospheric density models on the Stella satellite on 27 May 2015.

As shown in Table 4, DTM2000 is more suitable for orbit determination and prediction calculated using global laser ranging data at an orbital altitude of 815 km. Figure 5 shows a typical scenario.

**Figure 6:**
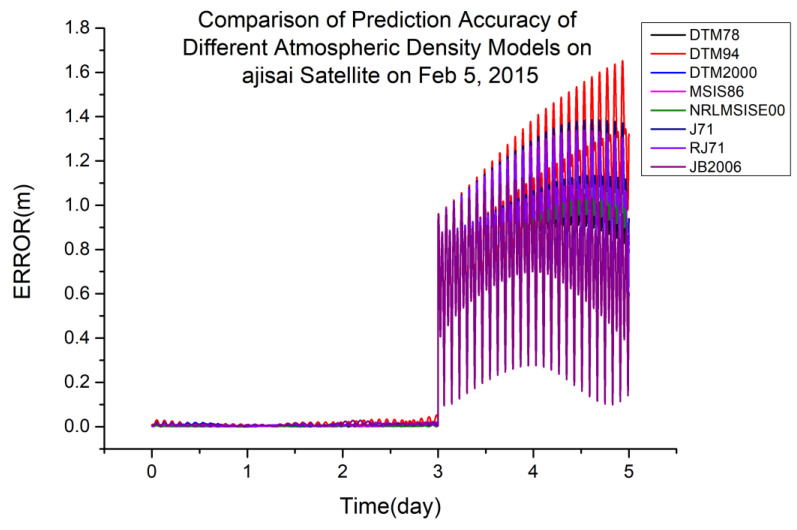
Comparison of the prediction accuracies of different atmospheric density models using the Ajisai satellite on 5 February 2015.

As shown in Table 4, JB2006 is more suitable for orbit determination and prediction calculated using global laser ranging data at an orbital altitude of 1490 km. Figure 6 shows a typical scenario.

To explore the impact of different atmospheric density models on the orbit determination and prediction calculated using single-station satellite laser ranging data, data from the Yarragadee Station in Australia (station number 7090) were selected for calculation. The station has a large time span and sufficient data points, which is conducive to orbit determination calculation. Using the same calculation method as for the multi-station data, single-station ranging data were calculated. Table 3 shows the results of the orbit prediction accuracy ranking using station 7090 laser ranging data in 2015.

From Table 5, it is not difficult to see that the calculation accuracies of the different atmospheric density models calculated using single-station laser ranging data varied with different orbital altitudes. The representative accuracy calculation results from the 2015 data are taken and plotted below.

**Figure 7:**
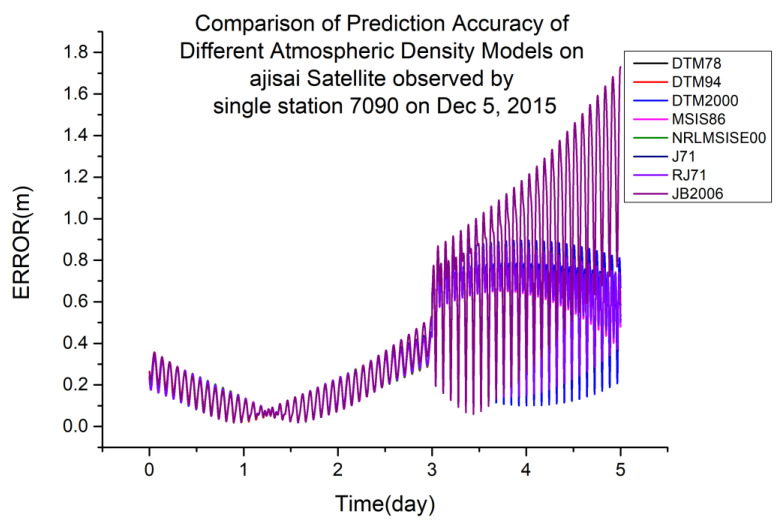
Comparison of prediction accuracies of different atmospheric density models using the Ajisai satellite, observed from a single station (7090) on 5 December 2015.

As shown in Table 5, MSIS86 is more suitable for orbit determination and prediction calculated using single-station laser ranging data at an orbital altitude of 1490 km. Figure 7 shows a typical scenario.

**Figure 8:**
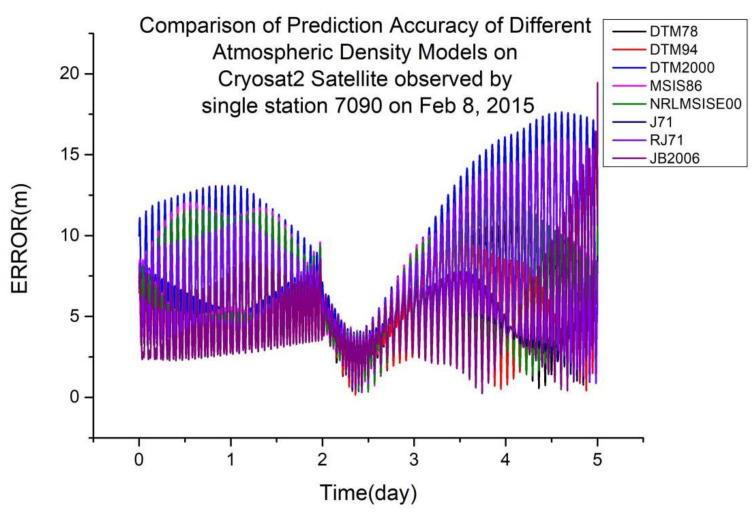
Comparison of prediction accuracies of different atmospheric density models using the Cryosat2 satellite, observed from a single station (7090) on 8 February 2015.

As shown in Table 5, JB2006 is more suitable for orbit determination and prediction calculated using single-station laser ranging data at an orbital altitude of 720 km. Figure 8 shows a typical scenario.

**Figure 9:**
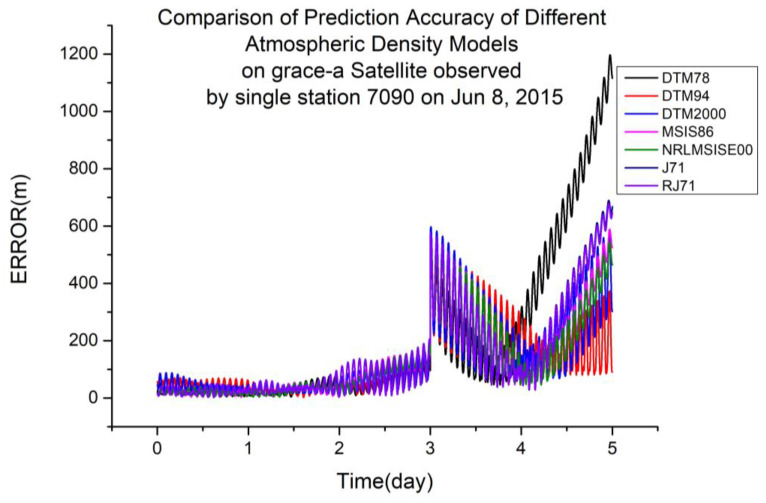
Comparison of prediction accuracies of different atmospheric density models using the GRACE-A satellite, observed from a single station (7090) on 8 June 2015.

As shown in Table 5, DTM94 is more suitable for orbit determination and prediction calculated using single-station laser ranging data at an orbital altitude of 485 km. Figure 9 shows a typical scenario.

## 5. Discussion

Table 6 shows the results of summarizing the scores of the same atmospheric density model. Table 6 summarizes the scores of various atmospheric densities in different situations in Table 4 and Table 5. Taking DTM2000 for example, this score is the sum of the data in the DTM2000 column of Table 4 and the data in the DTM2000 column of Table 5. The scores of each atmospheric model are arranged from high to low in Table 6. It is not difficult to see that the DTM2000 atmospheric density model has the highest error prediction score, followed by the RJ71 model, and the JB2006 model has the lowest score.

## 6. Conclusions

To quantitatively evaluate the impact of different atmospheric density models on the orbit prediction calculations, we proposed a scoring method based on the extrapolation accuracy of different atmospheric density models at different orbit altitudes. This method is based on published space-target laser ranging data, which are used to determine the orbits of space targets at different orbital altitudes and use different atmospheric density models for orbit prediction. The prediction accuracies of specific time periods were ranked and scored from highest to lowest and then aggregated to obtain a quantitative atmospheric density model orbit prediction accuracy scoring table.

The results from Table 4 and Table 5 show the following:

- The advantages and disadvantages of atmospheric density models vary at different orbital altitudes. For multi-station laser ranging data, the best-performing atmospheric density models at an altitude of 425 km are DTM2000 and RJ71; the best-performing atmospheric density model at an altitude of 485 km is NRLMSISE00; the best-performing atmospheric density model at an altitude of 720 km is JB2006; the best-performing atmospheric density model at an altitude of 815 km is DTM2000; and the best-performing atmospheric density model at an altitude of 1490 km is JB2006.
- For single-station laser ranging data from station number 7090, the best-performing atmospheric density model at an altitude of 485 km is DTM94; the best-performing atmospheric density model at an altitude of 720 km is JB2006; and the best-performing atmospheric density model at an altitude of 1490 km is MSIS86.

Summarizing the scores shows that the DTM2000 atmospheric density model is the best for orbit prediction calculations of low-Earth-orbit space debris, which is retired satellites with small area–mass ratios. The RJ71 model and the NRLMSISE00 model followed closely in terms of scores. The difference in scores between these three models is not very significant, and using these three models for low-Earth-orbit prediction error results will be more stable.

## Figures and Tables

**Table 1:** Selection of space debris for the experimental target.

Name	NORADID	Apogee/km	Orbit	Inclination/°	Windward Area/m^2^	Mass/kg
SpinSat	40,314	425	Circle	51.6	0.2452	52.65
GRACE-A	27,391	485	Circle	89	1.005~1.06	432
CryoSat-2	36,508	720	Circle	92	1.5648	711
Stella	22,824	804	Near Circle	98.6	0.4524	48
Ajisai	16,908	1490	Circle	50	3.6305	685

**Table 2:** Prediction error of SpinSat using different atmospheric density models in 2015.

Day of Year	DTM78 (m)	DTM94 (m)	DTM2000 (m)	J71 (m)	RJ71 (m)	MSIS86 (m)	NRLMSISE00 (m)	JB2006 (m)
4	1931.600133	3079.920804	1458.919706	2116.757042	2079.199273	1763.564414	1947.077307	None
7	2413.552003	1449.132559	7373.937369	2428.190075	2541.417706	5225.252596	4168.514523	None
10	6523.702123	4318.961777	6975.571103	7509.519426	7584.702847	6534.514692	6453.941776	None
13	878.6535121	1569.094311	186.8271987	1168.365429	1184.146046	348.8813334	732.8545937	None
16	4732.151926	1248.865147	3901.123424	4848.155709	4833.616385	4067.938681	4417.50813	None
19	5333.990435	1918.635851	3617.147013	5489.835638	5448.790281	4725.461779	4862.629035	None
22	1195.261982	3492.042957	2231.433994	1507.844551	1534.013338	1402.649516	1546.57513	None
25	2774.171141	1602.739092	2233.519238	2363.856728	2344.851527	2760.252276	2477.83181	None
28	3569.166135	3059.533481	2987.73538	3768.047339	3764.942462	3380.587181	3582.752166	None
31	3743.680387	1925.404499	2369.868958	3689.234171	3658.263772	3411.715633	3373.188497	None
34	4704.853081	2050.631091	2429.282375	3981.901854	3927.73963	4354.482803	4091.370207	None
37	1220.040145	407.3317976	377.0501051	179.8727984	165.5032465	671.248745	511.4899832	None
46	1831.734102	889.4722385	1495.626767	1970.517863	1926.734394	1743.008323	1811.290293	None
49	7680.866673	8635.615291	8246.155763	7455.445173	7511.144962	7610.631855	7632.910916	None
52	2597.16714	1569.410387	1605.946106	2860.033677	2790.721418	2703.001168	2687.572104	None
55	7656.145474	8490.292897	8117.931219	7541.922492	7558.769213	7581.568843	7560.633819	None
85	2360.890646	2459.110409	3800.377898	2652.62701	2691.752874	2253.842598	2335.697072	None
100	11,636.09167	10,427.33168	11,430.33493	11,445.45271	11,421.74373	11,604.20247	11,547.13792	None
109	11,424.47437	9916.908893	8437.244075	11,491.33912	11,452.2517	10,836.0404	10,916.44246	None
112	7181.635438	9147.320387	10,179.04015	8196.492788	8227.888443	7768.549827	7818.857253	None
115	12,757.81725	10,045.68352	7822.872368	11,273.22851	11,247.70294	12,250.68636	12,074.74769	None
124	932.5633444	814.011423	1349.889184	1265.836975	1240.740571	737.3913414	642.4145322	None
142	15,895.02702	17,169.73294	14,002.43708	15,879.05673	15,799.69467	15,280.89231	15,263.56853	None
145	830.95509	1968.245401	2114.269351	994.4573895	1004.565394	1002.741409	940.1047626	None
148	581.2062897	642.9255554	2177.784802	629.8109069	629.9201598	704.3030868	657.0174016	None
151	1211.973606	988.409299	556.9590948	931.7087737	947.4996212	1072.230125	1038.708581	None
154	789.1508336	619.5120045	352.9180023	604.809844	643.0717759	840.0688151	672.8206851	None
157	2883.022325	2525.966774	3323.514894	3009.237086	2964.038334	2692.66321	2863.215203	None
160	11,798.37263	11,128.4576	11,517.00753	11,835.79413	11,792.88253	11,909.58912	11,889.76281	None
163	1889.706197	1766.398949	2484.120815	1703.919178	1718.600351	1389.751668	1694.680473	None
166	3758.600669	5197.474592	1698.531248	3469.507424	3468.187086	4570.164283	4267.055289	None
178	6743.051	7119.971186	7569.97595	6305.745448	6316.016906	6805.619408	6677.656748	None
181	6793.72063	6051.937709	5905.723973	6953.320515	6946.827446	6683.637648	6704.240135	None
190	362.4262961	504.1228635	573.7563068	138.1085118	140.5458193	397.175753	243.0272944	None
199	4058.895637	3380.960822	3700.687258	4045.152743	3995.112412	3936.560189	3894.248654	None
202	2693.146161	1725.500802	2405.482228	2583.293282	2548.014167	2253.71296	2272.572876	None
205	3768.638487	4312.106629	3708.680637	3883.418707	3886.545139	3795.290052	3728.824543	None
208	1627.227411	1878.738075	1024.786763	1673.68751	1670.832731	1579.634453	1533.613801	None
211	1180.538253	806.5945208	1820.368453	824.4971614	846.3165552	1335.26865	1222.439084	None
214	354.7834503	498.5327752	796.0710677	365.5853363	355.1858976	677.7189817	479.4927719	None
217	2982.863162	2943.435226	3015.421628	2829.626528	2865.154406	3215.472864	3131.683215	None
220	5280.901633	4798.360784	5471.066765	4884.023698	4850.413792	4764.003451	4712.07914	None
223	5841.227621	5440.541323	5389.325079	6247.024148	6222.983625	6294.259962	6573.475359	None
232	2464.611291	2945.164304	2890.109141	2556.961499	2569.458218	2516.085143	2543.160569	None
235	2462.61968	2768.264944	2783.260805	2531.838449	2544.492688	2303.792588	2442.334603	None
238	3525.873948	3249.666221	3284.946579	3570.243744	3567.128888	3324.488449	3395.378844	None
241	4290.110658	5908.756547	6039.836157	4571.28022	4595.245094	5396.097679	5247.266835	None
244	445.0045812	1039.234089	1187.321279	631.2764643	645.777949	711.9517264	748.6355443	None
247	5188.714791	4664.543176	4446.207533	4775.978815	4761.820946	4772.874603	4743.768285	None
250	962.5071078	915.6541998	751.6987707	709.7216693	708.1754737	794.7870124	781.5375739	None
277	4415.028285	6162.817672	5850.337504	4946.86744	4984.38439	4723.122808	4796.552915	None
280	3479.326832	2862.906281	3141.925644	3194.272299	3182.501624	3370.330644	3324.178701	None
283	14,380.75618	14,919.46064	14,254.47307	14,973.85354	14,972.03744	14,564.98197	14,610.56452	None
286	7715.086151	7684.165182	8269.420855	7535.406173	7550.867783	7720.544424	7712.679961	None
289	1853.708908	1562.230413	3254.182394	1687.526303	1736.083023	1858.457603	1898.565426	None
292	12,071.70545	11,193.74813	14,047.09718	11,459.18475	11,529.01707	12,072.6877	12,147.0764	None
295	2366.193566	1287.840352	4650.255211	2302.487535	2283.016581	2423.567814	2321.464863	None
298	3455.771606	4916.251338	312.1307575	3667.102817	3671.81249	3279.71035	3429.087875	None
301	2654.726524	3442.258596	1140.981472	2565.748038	2599.176146	2874.191043	2839.872177	None
304	2620.823237	2773.493033	2152.471575	3050.380065	2997.998836	2444.734105	2566.006423	None
307	4526.15703	5529.274445	6878.556217	4498.979455	4582.189076	4729.666843	4455.28715	None
319	7820.199407	9510.78637	10083.96285	8586.681294	8611.426113	8959.493111	8762.753366	None
322	3619.487502	3064.643892	616.4473538	2438.125387	2418.368213	2749.788236	2619.950892	None
325	8792.96411	9704.18663	4119.912245	7680.191562	7665.423885	8328.169259	7986.019216	None
328	3449.395612	6271.79717	279.4746186	2497.473109	2518.442123	3601.928059	3365.094898	None
331	217.9783104	2910.17943	3312.678835	861.1899993	835.6972508	180.4582137	168.7785088	None
334	941.8044225	1786.292102	2007.639369	1247.93625	1210.451103	963.2524674	733.4894426	None
337	3680.017576	4334.610395	3531.894377	3114.393267	3121.323663	3219.553245	3405.502959	None
340	9437.666525	8727.028693	7116.148402	9319.746918	9313.644902	9809.397596	9664.515033	None
343	4884.999263	5503.695338	7342.026747	4839.368191	4817.709983	4684.456979	4677.949205	None
346	6950.305951	7938.067944	4047.450982	6649.403855	6703.780536	6794.532829	6973.470208	None
349	11,322.25148	10,726.56432	10,350.80818	11,221.36822	11,278.78786	11,288.75473	11,397.6071	None
352	7371.244204	8101.553627	6221.025583	7244.05491	7208.557565	7186.48702	7277.367959	None

**Table 3:** Table of scores for the 1-day prediction error of SpinSat using different atmospheric density models in 2015.

Day of Year	DTM78	DTM94	DTM2000	J71	RJ71	MSIS86	NRLMSISE00	JB2006
4	6	2	8	3	4	7	5	1
7	7	8	2	6	5	3	4	1
10	6	8	4	3	2	5	7	1
13	5	2	8	4	3	7	6	1
16	4	8	7	2	3	6	5	1
19	4	8	7	2	3	6	5	1
22	8	2	3	6	5	7	4	1
25	2	8	7	5	6	3	4	1
28	5	7	8	2	3	6	4	1
31	2	8	7	3	4	5	6	1
34	2	8	7	5	6	3	4	1
37	2	5	6	7	8	3	4	1
46	4	8	7	2	3	6	5	1
49	4	2	3	8	7	6	5	1
52	6	8	7	2	3	4	5	1
55	4	2	3	8	7	5	6	1
85	6	5	2	4	3	8	7	1
100	2	8	6	5	7	3	4	1
109	4	7	8	2	3	6	5	1
112	8	3	2	5	4	7	6	1
115	2	7	8	5	6	3	4	1
124	5	6	2	3	4	7	8	1
142	3	2	8	4	5	6	7	1
145	8	3	2	6	4	5	7	1
148	8	5	2	7	6	3	4	1
151	2	5	8	7	6	3	4	1
154	3	6	8	7	5	2	4	1
157	5	8	2	3	4	7	6	1
160	5	8	7	4	6	2	3	1
163	3	4	2	6	5	8	7	1
166	5	2	8	6	7	3	4	1
178	5	3	2	8	7	4	6	1
181	4	7	8	2	3	6	5	1
190	5	3	2	8	7	4	6	1
199	2	8	7	3	4	5	6	1
202	2	8	5	3	4	7	6	1
205	6	2	8	4	3	5	7	1
208	5	2	8	3	4	6	7	1
211	5	8	2	7	6	3	4	1
214	8	4	2	6	7	3	5	1
217	5	6	4	8	7	2	3	1
220	3	6	2	4	5	7	8	1
223	6	7	8	4	5	3	2	1
232	8	2	3	5	4	7	6	1
235	6	3	2	5	4	8	7	1
238	4	8	7	2	3	6	5	1
241	8	3	2	7	6	4	5	1
244	8	3	2	7	6	5	4	1
247	2	7	8	3	5	4	6	1
250	2	3	6	7	8	4	5	1
277	8	2	3	5	4	7	6	1
280	2	8	7	5	6	3	4	1
283	7	4	8	2	3	6	5	1
286	4	6	2	8	7	3	5	1
289	5	8	2	7	6	4	3	1
292	5	8	2	7	6	4	3	1
295	4	8	2	6	7	3	5	1
298	5	2	8	4	3	7	6	1
301	5	2	8	7	6	3	4	1
304	5	4	8	2	3	7	6	1
307	6	3	2	7	5	4	8	1
319	8	3	2	7	6	4	5	1
322	2	3	8	6	7	4	5	1
325	3	2	8	6	7	4	5	1
328	4	2	8	7	6	3	5	1
331	6	3	2	4	5	7	8	1
334	7	3	2	4	5	6	8	1
337	3	2	4	8	7	6	5	1
340	4	7	8	5	6	2	3	1
343	4	3	2	5	6	7	8	1
346	4	2	8	7	6	5	3	1
349	3	7	8	6	5	4	2	1
352	3	2	8	5	6	7	4	1

**Table 4:** One-day prediction error scores for different atmospheric density models at different orbital altitudes in 2015.

	Model	DTM78	DTM94	DTM2000	MSIS86	NRLMSISE00	J71	RJ71	JB2006
Target									
SpinSat (425 km)		341	360	377	368	373	358	378	73
GRACE-A (425 km)		493	486	499	500	515	501	499	179
CryoSat2 (720 km)		471	472	499	473	486	519	500	540
Stella (804 km)		489	493	524	514	509	494	493	516
Ajisai (1490 km)		458	404	449	409	435	423	457	493

**Table 5:** One-day prediction error scores for different atmospheric density models at different orbital altitudes observed by a single station (7090) in 2015.

	Model	DTM78	DTM94	DTM2000	MSIS86	NRLMSISE00	J71	RJ71	JB2006
Target									
GRACE-A (485 km)		358	391	376	373	384	360	366	154
CryoSat2 (720 km)		344	375	405	392	401	409	419	444
Ajisai (1490 km)		504	423	520	535	524	504	529	457

**Table 6:** Prediction error scores for different atmospheric density models.

Model	Score
DTM2000	3649
RJ71	3641
NRLMSISE00	3627
J71	3568
MSIS86	3564
DTM78	3458
DTM94	3404
JB2006	2856

## Data Availability

Not applicable.
